# Risk of major bleeding during extended oral anticoagulation in patients with first unprovoked venous thromboembolism: a systematic review and meta-analysis protocol

**DOI:** 10.1186/s13643-019-1175-5

**Published:** 2019-10-28

**Authors:** Faizan Khan, Miriam Kimpton, Tobias Tritschler, Grégoire Le Gal, Brian Hutton, Dean A. Fergusson, Marc A. Rodger

**Affiliations:** 1 School of Epidemiology and Public Health, University of Ottawa, Ottawa, Ontario Canada; 2 Clinical Epidemiology Program, Ottawa Hospital Research Institute, Ottawa, Ontario Canada; 3 Centre for Practice-Changing Research, The Ottawa Hospital, General Campus, 501 Smyth Road, Ottawa, Ontario K1H 8L6 Canada; 4 Ottawa Blood Disease Centre, Department of Medicine, The Ottawa Hospital, Ottawa, Ontario Canada

**Keywords:** Venous thromboembolism, Anticoagulation, Major bleeding

## Abstract

**Background:**

The optimal duration of anticoagulation after a first unprovoked venous thromboembolism (VTE) remains controversial. Deciding to stop or continue anticoagulant therapy indefinitely after completing 3 to 6 months of initial treatment requires balancing the long-term risk of recurrent VTE if anticoagulation is stopped against the long-term risk of major bleeding if anticoagulation is continued. However, knowledge of the long-term risk for major bleeding events during extended anticoagulation in this patient population is limited. We plan to conduct a systematic review and meta-analysis to quantify the risk for major bleeding events during extended oral anticoagulation in patients with first unprovoked VTE.

**Methods:**

Electronic databases including MEDLINE, EMBASE, and the Cochrane Central Register of Controlled Trials will be systematically searched with the assistance of an information specialist (from inception to March 1, 2019) to identify randomized controlled trials and prospective cohort studies reporting major bleeding during extended oral anticoagulation in patients with first unprovoked VTE, who have completed at least 3 months of initial anticoagulant therapy. Study selection, risk of bias assessment, and data extraction will be performed independently by at least two investigators. The number of major bleeding events and person-years of follow-up will be used to calculate the rate (events per 100 person-years) with its 95% confidence interval for each study cohort, during clinically relevant time periods of extended anticoagulant therapy. Results will be pooled using random effect meta-analysis.

**Discussion:**

The planned systematic review and meta-analysis will provide reliable estimates of the risk for major bleeding events during extended anticoagulation. This information will help inform patient prognosis and assist clinicians with balancing the risks and benefits of treatment to guide management of unprovoked VTE.

**Systematic review registration:**

PROSPERO CRD42019128597.

## Background

Venous thromboembolism (VTE), defined as deep vein thrombosis (DVT) or pulmonary embolism (PE), is a common, potentially fatal, yet treatable condition. Anticoagulation is the mainstay of VTE treatment and is divided into three phases: the initial phase (first 5 to 10 days after diagnosis), the long-term phase (from the initial phase to 3–6 months), and the extended phase (beyond 3–6 months) [1]. Anticoagulant therapy for at least 3 months is recommended in all patients with acute VTE [1]. Thereafter, balancing the long-term risk of recurrent VTE, defined by etiology of the VTE and the presence of persistent risk factors, against the long-term risk of major bleeding from anticoagulation informs treatment duration. Etiology of VTE is defined as provoked or unprovoked [2]. Patients with an unprovoked event have a high risk of recurrent VTE after discontinuation of anticoagulation (10% after 1 year, 36% after 10 years) [3]. Therefore, the 2016 American College of Chest Physicians and the 2014 European Society of Cardiology guidelines both suggest indefinite anticoagulation in patients with unprovoked VTE, unless they have a high risk of bleeding [1, 4].

A previous meta-analysis by Linkins and colleagues [5] reported a major bleeding rate of approximately 3% per year during extended anticoagulation. However, the analysis was based on a heterogeneous population of VTE patients (i.e., mix of provoked and unprovoked VTE), and the duration of extended anticoagulation in most included studies was limited to 3 months (i.e., 6 months of total treatment duration). To inform the decision on whether patients with a first unprovoked VTE should continue anticoagulation indefinitely, understanding the *long-term* risk of major bleeding on extended anticoagulation is crucial.

Since the publication of the meta-analysis by Linkins et al. in 2003, numerous prospective studies involving patients with a first unprovoked VTE have reported on the risk of major bleeding during extended anticoagulation with patient follow-up lasting beyond 3 months and up to a maximum follow-up of 4 years [6]. This provides the opportunity to summarize and establish reliable and precise estimates of the *long-term* risk of major bleeding on extended oral anticoagulant therapy in patients with first unprovoked VTE.

## Objective

The aim of this systematic review and meta-analysis is to quantify the rate of major bleeding events during extended oral anticoagulation in patients with a first unprovoked VTE, who have completed at least 3 months of initial treatment.

## Methods

This protocol was developed following the Preferred Reporting Items for Systematic Review and Meta-Analysis Protocols (PRISMA-P) statement [7] and is registered in the PROSPERO international register of prospective systematic review database (CRD42019128597). Any modifications made to the study methods during conduct of the review will be reported and justified in the publication of the final report. The final publication will be reported according to guidance from the PRISMA statement [8].

### Eligibility criteria

Studies meeting the following criteria will be included in the systematic review.

#### Population

The targeted group of participants will be adults with a first episode of objectively confirmed, symptomatic major VTE (proximal DVT or PE) that is either unprovoked or provoked by minor transient risk factors, according to the International Society on Thrombosis and Hemostasis (ISTH) definition [2]. Patients will be eligible if they have received treatment with an approved oral anticoagulant therapy, continued for a minimum of six additional months beyond completion of at least 3 months of anticoagulation with either (1) intravenous heparin or low molecular weight heparin (LMWH) for 3 months, or (2) intravenous heparin or LMWH for at least 5 days followed by dabigatran, edoxaban, or a vitamin K antagonist, or (3) apixaban or rivaroxaban. Studies will be excluded if they only include patients with VTE associated with major transient and/or persistent provoking risk factors according to the ISTH definition [2].

#### Interventions and comparators

The review will include studies wherein participants have received treatment with an approved oral anticoagulant therapy, continued for a minimum of six additional months beyond completion of at least 3 months of anticoagulation. As the study objective is to establish the rate of major bleeding during extended anticoagulation, a comparator is not applicable. As such, all studies, including each arm of a randomized controlled trial (RCT), will be evaluated as an independent observational cohort, with follow-up starting at the time that oral anticoagulants are continued for secondary prevention (i.e., beyond completion of at least 3 months of *initial* treatment).

#### Outcome

The primary outcome will be the rate of major bleeding (as defined by the ISTH [9] or by individual studies) on extended oral anticoagulation.

#### Study design

Studies eligible for this systematic review will consist of both RCTs and prospective cohort studies. Case reports, case series, case-control, or cross-sectional studies, as well as retrospective cohort and registry-based studies, will be excluded.

### Search strategy

In conjunction with an information specialist, electronic databases including MEDLINE, EMBASE, and the Cochrane Central Register of Controlled Trials will be systematically searched (from inception to March 01, 2019) with no language restrictions. Search terms will be related to VTE, major bleeding, anticoagulants, and study design. We will use medical subject heading (MeSH) terms. We will supplement our search with keywords and adjust vocabulary and syntax across databases. The search strategy for EMBASE and MEDLINE was reviewed by an information specialist and is presented in Table 1 and Table 2, respectively. Reference lists of retrieved articles will be hand-searched to identify additional relevant studies, while gray literature will not be considered.

**Table 1:** EMBASE search strategy

No.	Searches
1	Venous Thrombosis/
2	(ven* adj2 thrombos*).ti, ab.
3	Deep Vein Thrombosis/
4	(deep adj3 thrombos*).ti, ab.
5	Pulmonary Embolism/
6	(pulmonary adj2 embolism*).ti, ab.
7	Venous Thromboembolism/
8	(ven* adj2 thromboembolism*).ti, ab.
9	Recurrent Venous Thromboembolism/
10	1 or 2 or 3 or 4 or 5 or 6 or 7 or 8 or 9
11	secondary prevention/
12	secondary prevention*.ti, ab.
13	(relapse adj2 prevention*).ti, ab.
14	(extended adj2 therap*).ti, ab.
15	11 or 12 or 13 or 14
16	Hemorrhage/
17	(hemorrhag* or haemorrhag*).ti, ab.
18	16 or 17
19	Anticoagulants/
20	Antithrombins/
21	(anticoagulant* or anti-coagulant* or antithrombin* or anti-thrombin*).tw.
22	(thrombin adj3 inhibit*).tw.
23	(Factor Xa adj2 (antagonist? or inhibit* or block*)).tw.
24	heparin/ or exp heparin, low-molecular-weight/
25	(heparin* or beparine or clarin or contusol or disebrin or eleparon or elheparin or elheparon or epiheparin or gag 98 or helberina or hepaflex or hepalean or heparitin* or hepcon or hepsal or inhepar or inviclot or lipo-hepin or lipohepin or liquemin or liquemine or menaven or monoparin or mucoitin or multiparin or nevparin or noparin or panheparin or panhepin or panheprin or parinix or praecivenin or pularin or thromb*or niparin or vetren or vaster).tw.
26	liquaemin.tw.
27	dalteparin*.tw.
28	fragmin*.tw.
29	enoxaparin*.tw.
30	clexane.tw.
31	lovenox.tw.
32	fraxiparin*.tw.
33	nadroparin*.tw.
34	Warfarin/
35	(warfarin or warfant or tedicumar or savaysa or endoxaban or befarin or adoisine or carfin or circuvit or coumadan or coumafene or coumaphene or dagonal or tintorane or uniwarfin or waran or warfar or warnerin or farin or jantoven or kumatox or maforan or orfarin or panwarfarin or panwarfin or prothromadin or warfil* or sofarin).tw.
36	coumadin*.tw.
37	aldocumar.tw.
38	marevan.tw.
39	(Vitamin K adj2 (antagonist? or inhibit* or block*)).tw.
40	19 or 20 or 21 or 22 or 23 or 24 or 25 or 26 or 27 or 28 or 29 or 30 or 31 or 32 or 33 or 34 or 35 or 36 or 37 or 38 or 39
41	(random$ or placebo$ or single blind$ or double blind$ or triple blind$).ti, ab.
42	RETRACTED ARTICLE/
43	or/41-42
44	(animal$ not human$).sh,hw.
45	(book or conference paper or editorial or letter or review).pt. not exp. randomized controlled trial/
46	(random sampl$ or random digit$ or random effect$ or random survey or random regression).ti, ab. not exp. randomized controlled trial/
47	43 not (44 or 45 or 46)
48	exp cohort analysis/
49	exp longitudinal study/
50	exp prospective study/
51	exp follow up/
52	cohort$.tw.
53	or/48–52
54	47 or 53
55	10 and 15 and 18 and 40 and 54
56	10 and 18 and 40 and 54

**Table 2:** MEDLINE search strategy

No.	Searches
1	exp Venous Thrombosis/
2	(ven* adj2 thrombos*).ti, ab.
3	exp Deep Vein Thrombosis/
4	(deep adj3 thrombos*).ti, ab.
5	exp Pulmonary Embolism/
6	(pulmonary adj2 embolism*).ti, ab.
7	Venous Thromboembolism/
8	(ven* adj2 thromboembolism*).ti, ab.
9	Recurrent Venous Thromboembolism/
10	1 or 2 or 3 or 4 or 5 or 6 or 7 or 8 or 9
11	secondary prevention/
12	secondary prevention*.ti, ab.
13	(relapse adj2 prevention*).ti, ab.
14	(extended adj2 therap*).ti, ab.
15	11 or 12 or 13 or 14
16	exp Hemorrhage/
17	(hemorrhag* or haemorrhag*).ti, ab.
18	16 or 17
19	Anticoagulants/
20	Antithrombins/
21	(anticoagulant* or anti-coagulant* or antithrombin* or anti-thrombin*).tw.
22	(thrombin adj3 inhibit*).tw.
23	(Factor Xa adj2 (antagonist? or inhibit* or block*)).tw.
24	heparin/ or exp. heparin, low-molecular-weight/
25	(heparin* or beparine or clarin or contusol or disebrin or eleparon or elheparin or elheparon or epiheparin or gag 98 or helberina or hepaflex or hepalean or heparitin* or hepcon or hepsal or inhepar or inviclot or lipo-hepin or lipohepin or liquemin or liquemine or menaven or monoparin or mucoitin or multiparin or nevparin or noparin or panheparin or panhepin or panheprin or parinix or praecivenin or pularin or thromb*or niparin or vetren or vaster).tw.
26	liquaemin.tw.
27	dalteparin*.tw.
28	fragmin*.tw.
29	enoxaparin*.tw.
30	clexane.tw.
31	lovenox.tw.
32	fraxiparin*.tw.
33	nadroparin*.tw.
34	Warfarin/
35	(warfarin or warfant or tedicumar or savaysa or endoxaban or befarin or adoisine or carfin or circuvit or coumadan or coumafene or coumaphene or dagonal or tintorane or uniwarfin or waran or warfar or warnerin or farin or jantoven or kumatox or maforan or orfarin or panwarfarin or panwarfin or prothromadin or warfil* or sofarin).tw.
36	coumadin*.tw.
37	aldocumar.tw.
38	marevan.tw.
39	(Vitamin K adj2 (antagonist? or inhibit* or block*)).tw.
40	19 or 20 or 21 or 22 or 23 or 24 or 25 or 26 or 27 or 28 or 29 or 30 or 31 or 32 or 33 or 34 or 35 or 36 or 37 or 38 or 39
41	randomized controlled trial.pt.
42	controlled clinical trial.pt.
43	random allocation.sh.
44	double-blind method.sh.
45	single-blind method.sh.
46	41 or 42 or 43 or 44 or 45
47	clinical trial.pt.
48	(clin$ adj25 trial$).ti, ab.
49	(singl$ or doubl$ or trebl$ or tripl$) adj25 (blind$ or mask$)).ti, ab.
50	placebos.sh.
51	placebo$.ti, ab.
52	random$.ti, ab.
53	research design.sh.
54	exp cohort studies/
55	cohort$.tw.
56	controlled clinical trial.pt.
57	epidemiologic methods/
58	48 or 49 or 50 or 51 or 52 or 53 or 54 or 55 or 56 or 57
59	46 or 58
60	10 and 15 and 18 and 40 and 59
61	10 and 18 and 40 and 59

### Data management and selection process

Results from the literature search will be uploaded to Covidence [10]. Screening of titles/abstracts as well as full-text articles will be performed by at least two independent investigators of the review team. Discrepancies will be resolved by consensus discussion or by a third person if needed. The process of study selection will be presented using the PRISMA flow diagram [8]. In the case of published reports including duplicate patients, only the publication with the longest follow-up of patients will be included.

### Data collection

Data from included studies will be collected using a standardized data extraction form. Data extraction will be conducted by at least two reviewers independently, with clarifications requested from the study’s authors when necessary. A third person will verify a subset of the studies to ensure accurate data collection. The standardized data extraction form will be piloted by two reviewers independently for the first five full-text articles. After comparing the results of the pilot phase for data extraction, further changes will be made to the standardized data extraction form, if needed. Disagreements between reviewers will be resolved by consensus or by a third person if required.

We will collect the following information from included studies:

- Study information: year of study publication, author, and journal information
- Study characteristics: design, period, country(ies), funding source, and criteria used to define VTE and its etiology.
- Participant characteristics: number of eligible patients, number of eligible male patients, mean age, site of initial VTE, type and dose of anticoagulant used during the initial treatment period, person-years of follow-up, and number of patients lost to follow-up.
- Intervention characteristics: type and dose of anticoagulant agent used during the extended treatment period.
- Outcomes: major, clinically relevant non-major, intracranial, and fatal bleeding (as defined by the ISTH or by individual studies), as well as recurrent VTE and recurrent fatal PE. Each study’s chosen definition of bleeding outcomes will be rigorously documented.

### Outcomes and prioritization

The primary outcome will be the rate of major bleeding during extended oral anticoagulation. Major bleeding is a patient-relevant outcome and the primary safety outcome of all recent phase III anticoagulation trials [11]. Secondary outcomes will include the rate of clinically relevant non-major bleeding, intracranial bleeding, fatal bleeding, recurrent VTE, and fatal recurrent PE during extended oral anticoagulation. We will also calculate the case-fatality rate of major bleeding and recurrent VTE during extending anticoagulation, from the total number of fatal (bleeding and recurrent PE events) divided by the total number of major bleeding and recurrent VTE events, respectively. Bleeding outcomes will be defined as per ISTH definition [9] for the primary analysis, and secondary analysis will include bleeding outcomes as defined by the individual studies. These secondary outcomes were chosen because of their clinical relevance and importance in assessing the net clinical benefit of extended anticoagulation.

### Risk of bias assessment

At least two reviewers will independently assess the risk of bias at the individual study level. For a subset of studies, a third reviewer will verify the accuracy of the risk of bias assessment done by the first two reviewers. Conflicts will be resolved by consensus or by a third reviewer if needed. Given each arm of a RCT will be evaluated as an independent observational cohort, the risk of bias for each study will be assessed using the Newcastle-Ottawa scale for prospective cohort studies [12].

### Data synthesis

The rate (expressed as events per 100 person-years) of the primary and secondary outcomes will be calculated from each observational cohort from the number of events and person-years of follow-up. If feasible from the available data, these rates will be calculated and reported at standardized time intervals during extended anticoagulation (i.e., at 6, 12, 18, 24, 36, 48, 60, and 120 months following completion of the *initial* 3- to 6-month period of anticoagulation) to account for the different length of follow-up from each study. To assess clinical and statistical heterogeneity, we will compare study design, patients’ characteristics, and studied interventions of included studies prior to pooling results. Statistical heterogeneity will be measured using Cochrane’s Q (statistically significant at *p* < 0.10) and the *I*^2^ statistic (> 75% considered to represent high heterogeneity).

If pooling is deemed appropriate, we will combine the total number of major bleeding events and person-years of follow-up across all included study cohorts to calculate a weighted estimate of the absolute rate of major bleeding per 100 person-years of follow-up. If and when possible, this pooled rate will be calculated for each of the standardized time periods defined above. Furthermore, the cumulative incidence of major bleeding events at each of the standardized time intervals will be estimated from the absolute rate of major bleeding events at each time period, if feasible from the available data. These analyses will be repeated for all secondary outcomes. A random effect model will be used for data synthesis due to the expected diversity of the studied interventions.

All meta-analyses will be performed using StatsDirect version 3 (Cheshire, UK) software [13].

#### Planned sensitivity analyses

Sensitivity analyses will be undertaken to establish the robustness of primary findings. We will perform the following sensitivity analyses excluding study cohorts whose event rates are outliers, as well as excluding studies judged to be at high risk of bias. Estimates from overall and sensitivity analyses will be compared to gauge the impact of potential heterogeneity and biases on the primary results.

#### Planned subgroup analyses

If feasible from the available data, subgroup analyses will be performed based on the study design (i.e., cohorts derived from randomized trials versus prospective observational studies), type of anticoagulation used for extended treatment, and patient characteristics. Several patient characteristics of interest were previously shown to be associated with bleeding in VTE patients receiving anticoagulation or used to define risk of recurrent VTE and case fatality in this patient population. Accordingly, we will include the following characteristics: sex [14–19], age (less than 65 years old and 65 years old or over) [1, 20–22], prior major bleeding [1, 20–26], concomitant use of anti-platelet therapy [1, 20], anemia (i.e., hemoglobin less than 90, 100, 110, 120, and 130 g/L) [23–27], creatinine clearance less than 30, 50, and 60 mL/min [1, 28], and site of initial VTE event (i.e., isolated proximal DVT, isolated PE, or DVT and PE) [29]. Findings from all analyses will be presented in the final report.

### Meta-bias assessment

Meta-bias will be assessed through the use of funnel plots to evaluate potential reporting bias if applicable. Additionally, we will compare a random effect estimates to fixed effect estimates to evaluate whether small sample bias is present. Finally, selective outcome reporting will be assessed by comparing the reported outcome from the included studies to their published protocol, where available.

## Discussion

In this systematic review and meta-analysis, we aim to provide reliable estimates regarding the risk of major bleeding events at clinically relevant time points during extended anticoagulation in patients with a first unprovoked VTE. This knowledge will inform clinical practice guidelines, and help clinicians and patients balance the risks and benefits of anticoagulation to guide treatment duration.

## Data Availability

Not applicable.

## Abbreviations

DVT: Deep vein thrombosis
ISTH: International Society on Thrombosis and Hemostasis
LMWH: Low molecular weight heparin
PE: Pulmonary embolism
PRISMA: Preferred Reporting Items for Systematic Reviews and Meta-analysis
PRISMA-P: Preferred Reporting Items for Systematic Reviews and Meta-Analysis Protocols
RCT: Randomized controlled trial
VTE: Venous thromboembolism
